# Assessing improvement capability in healthcare organisations: a qualitative study of healthcare regulatory agencies in the UK

**DOI:** 10.1093/intqhc/mzy085

**Published:** 2018-04-19

**Authors:** Joy Furnival, Ruth Boaden, Kieran Walshe

**Affiliations:** 1 Alliance Manchester Business School, Manchester, UK; 2 NHS Improvement, Wellington House, Waterloo Road, London, UK

**Keywords:** quality management, quality improvement < quality management, quality measurement < quality management, external quality assessment, certification/accreditation of hospitals < external quality assessment

## Abstract

**Objectives:**

Healthcare regulatory agencies are increasingly concerned not just with assessing the current performance of the organisations they regulate, but with assessing their improvement capability to predict their future performance trajectory. This study examines how improvement capability is conceptualised and assessed by healthcare UK regulatory agencies.

**Design:**

Qualitative analysis of data from six UK healthcare regulatory agencies was conducted. Three data sources were analysed using an *a priori* framework of eight dimensions of improvement capability identified from an extensive literature review.

**Setting:**

The focus of the research study was the regulation of hospital-based care, which accounts for the majority of UK healthcare expenditure. Six UK regulatory agencies that review hospital care participated.

**Participants:**

Data sources included interviews with regulatory staff (*n* = 48), policy documents (*n* = 90) and assessment reports (*n* = 30).

**Intervention:**

None—this was a qualitative, observational study.

**Results:**

This research study finds that of eight dimensions of improvement capability, process improvement and learning, and strategy and governance, dominate regulatory assessment practices. The dimension of service-user focus receives the least frequency of use. It may be that dimensions which are relatively easy to ‘measure’, such as documents for strategy and governance, dominate assessment processes, or there may be gaps in regulatory agencies’ assessment instruments, deficits of expertise in improvement capability, or practical difficulties in operationalising regulatory agency intentions to reliably assess improvement capability.

**Conclusions:**

The UK regulatory agencies seek to assess improvement capability to predict performance trajectories, but out of eight dimensions of improvement capability, two dominate assessment. Furthermore, the definition and meaning of assessment instruments requires development. This would strengthen the validity and reliability of agencies’ assessment, diagnosis and prediction of performance trajectories, and support development of more appropriate regulatory performance interventions.

## Introduction

Unexplained variations in healthcare performance continue to be a significant focus of public and political attention [1, 2]. In response to such variations, widespread concerns about patient safety [3, 4], and high-profile instances of failures in healthcare [5, 6], many governments have introduced or strengthened systems for formal oversight, accountability and regulation in healthcare [7, 8]. However, regulatory agencies themselves have often faced criticisms that their regulatory methods or regimes are not able to assess performance or quality accurately, or to diagnose and intervene to improve performance and quality effectively [9, 10]. In addition, the costs and benefits of regulation have also been questioned [11].

In response to such criticisms, some regulatory agencies have sought to move beyond directly assuring organisational performance or quality of care through mechanisms such as inspection and assessment, and are implementing programmes to strengthen the underlying organisational characteristics for organisations to develop and sustain their own improvement programmes through improvement approaches. In the wider academic literature, these characteristics are termed ‘improvement capability’, defined as the ‘organisational ability to intentionally and systematically use improvement approaches, methods and practices, to change processes and products/services to generate improved performance’ [12, p3]. This builds on a dynamic capabilities view, which suggests that organisational performance is driven through bundles of routines, described as distinctive capabilities, that are used to purposefully create and modify resources and routines that are contingent on local circumstances [13, 14]. However, the tacit nature of capabilities creates significant barriers to imitation, substitution or assessment [15]. A comprehensive literature review identified eight dimensions of improvement capability to support its assessment and development (Table 1).

**Table 1:** Dimensions of improvement capability [12]

Coding dimension	Description
Organisational culture	The core values, attitudes and norms and underlying ideologies and assumptions within an organisation
Data and performance	The use of data and analysis methods to support improvement activity
Employee commitment	The level of commitment and motivation of employees for improvement
Leadership commitment	The support by formal organisational leaders for improvement and performance
Service-user focus	The identification and meeting of current and emergent needs and expectations for service users
Process improvement and learning	Systematic methods and processes used within an organisation to make improvements through ongoing experimentation and reflection
Stakeholder and supplier focus	The extent of the relationships, integration and goal alignment between the organisation and stakeholders such as public interest groups, suppliers and regulatory agencies
Strategy and governance	The process in which organisational aims are implemented and managed through policies, plans and objectives

For regulatory agencies, assessing improvement capability may be important for two reasons. First, it may provide greater assurance about current performance; regulatory agencies can only undertake limited direct assessments of the quality of care, but may take some assurance that organisations with relatively higher improvement capability can monitor and assure quality for themselves. Second, improvement capability may have some value in predicting future performance, especially if problems with the quality of care are found. Organisations with extensive improvement capability may be more able to deal with such problems and bring about improvement for themselves, while those with limited improvement capability may need external support and intervention. More fundamentally, by focusing on assessing improvement capability, regulatory and improvement agencies are likely to encourage healthcare organisations themselves to pay greater attention to how they build and sustain improvement capability.

There are six regulatory agencies (Since this research was completed Monitor and the TDA have become a part of the same organisation with the operational name of NHS Improvement.) overseeing the healthcare system across the four countries of the UK, with several incorporating some assessment of improvement capability into their inspection, assessment or oversight regimes. However, there is little published literature that has examined how regulatory and improvement agencies can assess improvement capability within organisations. This research study examines how improvement capability is conceptualised and assessed in practice by healthcare regulatory agencies in the UK.

## Methods

The focus of the research study was the regulation of hospital-based care, which accounts for the majority of UK healthcare expenditure. Thus, the six healthcare regulatory agencies based in the UK that have responsibility for the oversight of hospital care were selected for the research study and all agreed to participate. These are the Care Quality Commission (CQC), Monitor, and the Trust Development Authority (TDA) in UK; Healthcare Improvement Scotland (HIS); Healthcare Inspectorate Wales (HIW) and the Regulatory and Quality Improvement Authority (RQIA) in Northern Ireland.

Qualitative methods offer an effective, flexible and common approach for data gathering. Three data sources from the agencies were used: policy documents, interviews and assessment reports (Table 2).

**Table 2:** Data sources and sample size

Agency	Documents	Interviews	Assessment reports
Time period	2010–15	2014–15	2013–15
HIS	18	8	5
HIW	13	9	5
RQIA	17	9	5
CQC	13	8	5
Monitor	16	7	5
TDA	13	7	5
Total	90	48	30

In order to understand how regulatory agencies define and conceptualise improvement capability and expressed intentions, published policy documents were identified, including agency strategies, operational plans and annual reports (*n* = 90). Following ethical approval to proceed, directors of policy, strategy or regulation within the agencies were contacted to take part in the research study and they aided the identification of suitable interview participants. About 7–9 interviews were held per agency (*n* = 48) representing a cross-section of clinical and non-clinical employees, including board-level roles, back office support and inspectors. Interviews were conducted face-to-face or via the telephone between October 2014 and April 2015; participation was voluntary and confidential. A semi-structured interview framework was used to examine agency purpose, intent, roles, methods and their understanding and assessment of improvement capability. Testing of the questions took place through pilot interviews. Interviews were recorded, anonymised and transcribed, and verbatim transcriptions were shared with participants to clarify any inaccuracies in the recordings. A few amendments were requested and were largely limited to grammar improvements and clarifications to recording problems.

Finally, five assessment reports per agency were selected (*n* = 30). The selection criteria required that they were publicly available, they represented a range of organisational performance, or they were referred to by interview participants as specific examples related to improvement capability. Two agencies in the sample do not routinely publish the results of their assessment processes; instead assessment reports for these agencies were identified through a detailed review of board reports from the agencies and regulated organisations. This may have influenced the extent to which the sample collected was representative of the range of organisational performance assessed.

Policy documents, interview transcripts and assessment report texts were loaded into electronic qualitative analysis software (NVivo10), and the eight dimensions of improvement capability (Table 1) were used as an *a priori* coding template to support content and thematic analysis of the policy documents, interviews and assessment reports [16, 17]. The combination of sources allowed a comparison of agencies’ expressed intent with practice. Coding consistency was reviewed using NVivo10 functionality to compare coding density and frequency across data sources [18].

## Results

This section begins by describing the UK agencies’ aims for improvement capability. It then discusses the content analysis of the data sources using the identified improvement capability dimensions, comparing agencies where appropriate. Following this, analysis themes are discussed.

### Aims of regulatory and improvement agencies

Analysis of agency policy documents identifies that agencies express intentions to strengthen improvement capability, with some agencies more explicit in their aims than others (Table 3). HIS and Monitor have specific strategic aims linked to developing improvement and associated capability in the National Health Service (NHS) in Scotland and England, respectively. Other agencies were less explicit, such as in Northern Ireland and Wales where a governmental aim to build capability exists rather than a specific one as within RQIA and HIW.

**Table 3:** UK regulatory agencies and associated improvement capability aims

UK country and population	Agency	Remit	Improvement capability aim
Scotland: 5.3 M	HIS	To advance improvement in healthcare in Scotland, and to support providers to deliver safer, more effective, person-centred care.	‘[Our / The QI Hub’s] purpose is to support NHS Scotland to develop the capacity, capability and infrastructures to excel in quality improvement with the aim of delivering the highest standards of care.’ [19]
Wales: 3 M	HIW	To inspect health boards and trusts, and regulate independent healthcare providers, general Practitioner practices, pharmacies and dental practices.	‘Fundamentals of care – Standard 6 ‘Participating in quality improvement activities, organisations and services reduce waste, variation and harm by […].’ [20]
Northern Ireland: 1.8 M	RQIA	To provide independent assurance about the quality of health and social care services.	‘We will promote the use of accredited improvement techniques and ensure that there is sufficient capacity and capability within the health and social care [system].’ [21](No explicit RQIA statement)
England: 53 M	CQC	To ensure health and social care services provide people with high quality care, and to encourage improvement.	‘[Our purpose is to] make sure health and social care services provide people with safe, effective, compassionate, high-quality care and we encourage care services to improve.’ [22]
England: ~149 Foundation Trusts	Monitor	To authorise, monitor and regulate foundation trust finances, quality and performance including price setting, preventing anti-competitive behaviour, whilst promoting care integration and protecting health services if providers become unsustainable.	‘We will focus in particular on the capabilities that drive long-term performance: […] We will also place more weight on the assessment of these capabilities […].’ [23]
England: ~90 Non-Foundation Trusts	TDA	To provide the oversight, scrutiny, and performance management of non-foundation trusts on behalf of the Department of Health and develop them into foundation trusts.	‘We want more than ever to focus on support and development …Our assessment of the credibility of plans, will focus on five broad areas [… including] leadership capability’ [24]

### Content analysis

**Figure 1:**
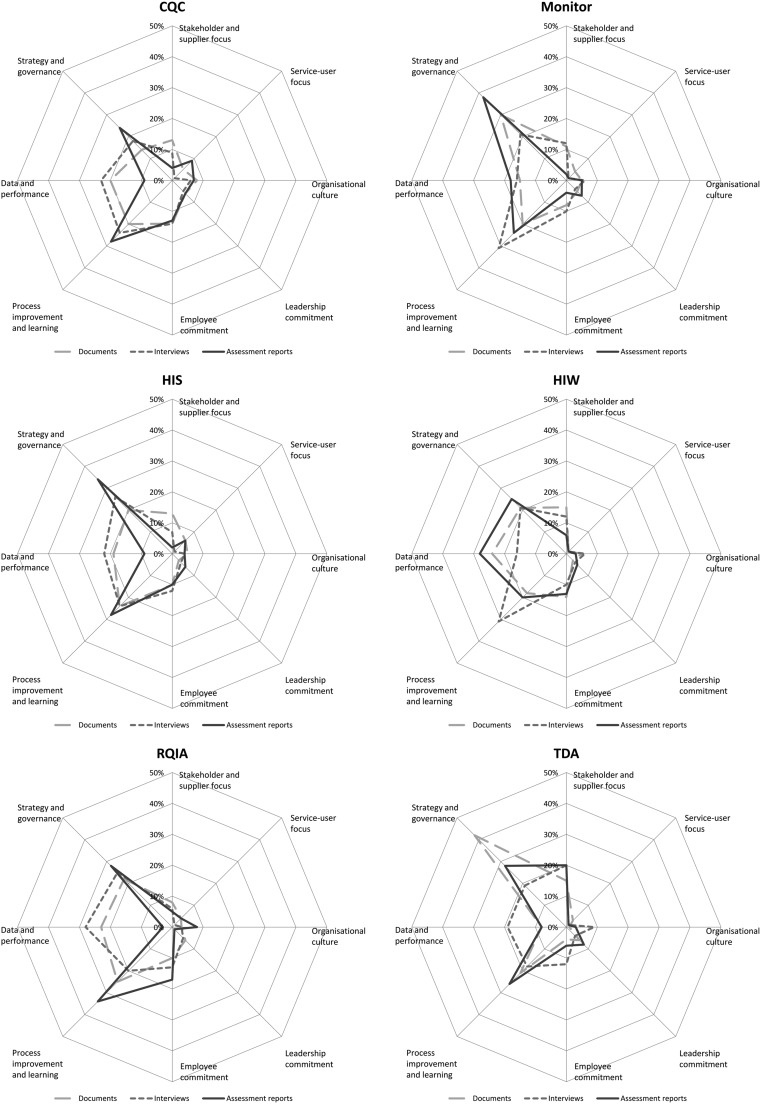
Content analysis.

Figure 1 presents the content analysis of the assessment reports and compares the coding frequency of the improvement capability dimensions within agency policy documents, interviews and assessment reports.

Overall, the analysis revealed that the dimensions of process improvement and learning, and strategy and governance, were most frequently found. Other dimensions were found less frequently, with service-user focus being the least frequent, and this skewed pattern was relatively consistent across agencies and data sources.

Table 4 shows representative examples of quotations from across the dimensions and were selected to illustrate how the agencies conceptualise the dimensions across the three data sources. Table 4 highlights how each dimension is used by the different regulatory agencies, adding depth to the initial content analysis in Fig. 1. This also enabled the examination of coding consistency across the data sample and to understand why dimensions were coded with different frequencies. For example, ‘leadership’ is a named assessment criterion for three of the regulatory agencies (Monitor, TDA and CQC); however, the content analysis indicated a low frequency of coding to the leadership commitment dimension across the data sources compared to other dimensions. The coding was reviewed for consistency, indicating that whilst assessment reports used leadership commitment as a high-level criterion, specific leadership activities, such as developing plans or communicating widely, fall within other dimensions in the assessment reports in this analysis and was coded as such. Perhaps this is to be expected as leadership commitment can cover many different aspects, can be used as a ‘catch all’, and does not exist within a vacuum.

**Table 4:** Representative sample of quotations across the data sources and dimensions

	Policy documents	Interviews	Assessment reports
Organisational culture	‘The boards of NHS organisations have a critical role in leading a culture which promotes delivery of a high quality, high value service.’ (Achieving Excellence. The Quality Delivery Plan for the NHS in Wales, Welsh Assembly Government, 2012)	‘*How do you support staff and build that behaviour and culture? … How do we build that peer pressure, that social acceptability, the whole environment people work in? […] That’s the challenge for the board.’* (Interview participant E, HIW).	'The culture of the team working within the department was one of cohesiveness, with staff displaying a very high level of professionalism and enthusiasm for the work they did.’ (Report B, CQC)
	‘The planning guidance also covered a range of other areas in relation to building a safety culture.’ (Board Papers, Trust Development Authority, March 2015)	*‘We perhaps talk in random terms about the culture of the organisation but we really don’t get to grips with the culture of the organisation … I suppose there are some sources of information that we would look at but whether you really understand the culture of the organisation from that.’* (Interview participant C, Monitor).	‘The vast majority of staff we spoke with said that they were unable to understand how decisions were made and were also unable to consistently describe to us the lines of accountability. There was a strong and consistent reference to a dysfunctional management structure and a ‘reactive culture’.’ (Report A, HIS)
Data and performance	‘Measurement is vital for both assurance and improvement. It must become second nature to all staff at all levels… The wider organisation and national level will look for assurance that outcomes are improving overall.’ (Achieving Excellence. The Quality Delivery Plan for the NHS in Wales, Welsh Assembly Government, 2012)	*‘[We look] at the data that we routinely have available to us, a range of different sources … which gives us a view of how trusts are performing … we would look at benchmarking them against each other, looking at both ends of the spectrum, both the good and the perhaps not so good.’* (Interview participant E, TDA)	‘The review team noted that the measures were self-reported by wards, but it also noted the lack of active challenge of the high reported compliance rates at Board level, even when related outcome measures were not improving.’ (HIS, Report C)
	‘The need for better access to benchmarking data was the most consistent development need identified […] the NHS TDA has developed its information provision and performance framework which includes a number of high level dashboards.’ (Delivering for Patients: the 2014/15 Accountability Framework for NHS Trust Boards, TDA, 2014)	*‘We look at from a board’s point of view, so what sort of targets are they setting the organisation, how are they tracking those and how can they demonstrate that they, as a board, have improved performance?’* (Interview participant E, Monitor)	‘Local outcomes were regularly audited and the trust was able to demonstrate how it had changed practice to improve results for patient’s year on year. The trust also benchmarked itself, and compared well against, national comparators.’ (CQC, Report B)
Employee commitment	‘RQIA will support and encourage individuals and organisations […] to be committed to the principles of best practice and continuous improvement.’ (Corporate Strategy. 2015–18, RQIA, 2015)	*‘If you haven’t got that ownership locally you… things are far more likely to succeed and it’s all […] just key principles in terms of ownership, engaging people, involving them in the change.’* (Interview participant F, HIS)	‘In the NHS 2013 Staff Survey, [X]% of staff reported that they felt satisfied with the quality of work and patient care they were able to deliver. This compared with a national average of [Y] %.’ (CQC, Report B)
	‘Most staff are aware change is necessary. They want to see how change will bring value and benefits to the people they care for. They also need to see how they can contribute to the changes, how their voice will be heard and, importantly, how they will be enabled to work differently in a way they know will bring about better, more quality-focused services to their patients and clients.’ (Together for Health. A Five-Year Vision for the NHS in Wales, Welsh Assembly Government, 2011)	*‘I would want to see that there’s a good connection between the board and the operational side of the organisation, that there’s strong clinical engagement, that they’re starting to just take ownership of some of the issues and get a grip of some of them.’* (HIW, Interview participant H)	‘We found that staff were committed to delivering good quality care and they were kind and caring, in many cases, we found issues with staff numbers, vacancies, resilience and skill mix.’ (HIW, Report B)
Leadership commitment	‘Strong and effective leadership within organisations from the ‘board to the ward’ is essential to drive improvement.’ (Delivering for Patients: the 2014/15 Accountability Framework for NHS Trust Boards, TDA, 2014)	*‘I’ve seen examples of really good strong leadership from individual people, like a chief executive or a director, a key lead within a service, but that […] again how sustainable is that, because if it’s one person driving everything, it’s not going to keep working.’* (Interview participant H, CQC).	‘There has been a lack of leadership within the organisation which has resulted in the failure to unite staff behind a common purpose.’ (Report A, HIS).
	‘Monitor has been tasked with making sure public providers of NHS care are well led, delivering quality care on a sustainable basis.’ (Monitor Annual Plan 2014–15, 2014)	*‘[One thing that gives] insight into the leadership is, we keep finding issues of inconsistency, so two wards right next to each other are very, very different. Two wards within the same specialty on different sites that are managed in completely different ways, and again, that begins to give you a sense of an insight into the quality of the leadership.’* (Interview participant D, HIW).	‘Strong managerial and professional leadership is required at all levels… There should be a shared commitment to making the system work. With strong leadership, it is easier to manage the tensions that arise.’ (Report B, RQIA).
Service-user focus	‘Our primary duty – and therefore our mission – focuses on patients…. Making a difference for patients will govern all that we do.’ (Monitor’s strategy 2014–17. Helping to redesign healthcare provision in England, Monitor, 2014a).	*‘We’ve got […] public and patient involvement (PPI) – and that’s going to drive the engagement of the service users [but organisations could not] engage with the person because that person had to be identified through the PPI office; and that just never happened.’* (Interview participant J, RQIA).	‘The Trust has a patient reference group, a consultative group including a number of patient representatives which allows the trust to hear feedback on the quality of its services and to consult with service users on proposed service developments.’ (Report D, TDA)
	‘CQC is on the side of people using health and social care services, their families and carers, highlighting where services are good and outstanding, and taking action where there is need for improvement.’ (Business plan. April 2015 to March 2016. An update to our three-year strategy: raising standards, putting people first, 2013–16, CQC, 2015)	*‘We talk about the fact that patients should be involved, and we encourage the trusts to involve the patients in their work, in their improvement work, in what goes on, in the scrutiny of what they’re doing, but we don’t actually have a large amount of public and patient involvement.’* (Interview participant G, TDA).	‘During some inspections, we observed the completion of a ‘This Is Me’ profile which captures important information about the person, their preferences and daily routines.’ (Report B, HIW)
Process improvement and learning	‘Opportunities for continuous learning by staff will be resourced and planned in order to continuously improve quality.’ (Quality 2020, Department of Health Services and Public Safety Northern Ireland, 2011)	*‘We require them to report in their annual governance statement on what their quality improvement methodology actually is.’* (Interview participant G, Monitor).	‘The trust encouraged innovation using recognised ‘Quality Improvement’ methodology. Approximately [X] consultants had undergone training in this and members of staff we spoke to were able to give examples of how they had been encouraged to drive change and improve their service.’ (Report E, CQC).
	‘One of the 10 ‘must do’ patient safety essentials is the use of the Scottish Patient Safety Programme care bundle for preventing infections when inserting and maintaining a peripheral vascular catheter.’ (Driving Improvement in the Quality of Healthcare. Annual Report 2013–14, HIS, 2014)	*‘We’ve tended to think there’s quite a lot of tools and methods out there and we probably don’t want to just…go in and say, we’re going to use the IHI model, then people will stop doing whatever else they’re doing.’* (Interview participant B, CQC)	‘There has been extensive training in quality improvement techniques including [X] staff trained in lean [and other methods].’ (Report C, HIS).
Stakeholder and supplier focus	‘These inspections look at how effectively […] services are working together and if they are delivering the best possible outcomes for the people who use those services.’ (Driving Improvement in the Quality of Healthcare. Annual Report 2013–14, HIS, 2014b)	*‘So, if you’re getting examples of … We’ve worked with the trust … we’ve achieved the following service developments, we’ve improved, you know, length of stay or whatever, that’s a real positive, you don’t get that a lot, but it’s something about the quality of their relationships and the track record.’* (Interview participant B, TDA)	‘There was evidence of limited engagement and communication between secondary and primary care. For improved patient outcomes, this is an area that needs to be reviewed and developed.’ (Report C, RQIA)
	‘It will be increasingly important for HIW […] to collaborate and coordinate their activities to scrutinise the performance of […] organisations to assess the quality of integrated care.’ (An Independent Review of the work of Healthcare Inspectorate Wales. The way ahead: to become an inspection and improvement body. Marks, 2014)	*‘How do they work with external providers, particularly community services and health visitors, and mental health… we actively check in two ways to make sure that they are working with and collaborating.’* (Interview participant F, CQC)	‘The Trust has involved stakeholders (e.g. governors, staff, CCG) in the development of quality objectives and associated plans.’ (Report A, Monitor)
Strategy and governance	‘Does the board have a credible strategy to provide quality, sustainable services to patients and is there a robust plan to deliver?’ (Well-led framework for governance reviews: guidance for NHS foundation trusts. Monitor, 2014b)	*‘[We] ask a Chief Exec what are the values of the organisation and what’s the strategic direction of the organisation, and they should be able to articulate that, and so should every member of staff that you speak with.’* (Interview participant D, CQC)	‘We are concerned that there is serious disconnect between the strategic planning processes and clinical and operational services… there is a mismatch in priorities and understanding regarding the practical delivery of clinical services and the development of strategic plans.’ (Report A, HIS).
	‘Our scrutiny activities include an ongoing programme to quality assure clinical governance processes and the quality of healthcare services provided across NHS boards in an impartial and objective way.’ (Our Strategy, 2014–20. HIS, 2014)	*‘I’m not saying I’ve no interest in the policy, but the policy is not what I’m interested in; it’s how it’s carried out on the ground that I’m interested in.’* (Interview participant E, RQIA)	‘Staff with governance responsibilities can explain how this works, and front-line staff know how to raise concerns. While there are a number of examples of issues being escalated, and acted upon, we have not seen evidence of a robust and systematic escalation process from ward to Board.’ (Report A, Monitor)

Three themes emerge from the analysis of the assessment of improvement capability: conceptualisation, assessment data and assessment practices.

### Conceptualisations

The first theme identified is that it was problematic to define, conceptualise and operationalise improvement capability. For example, policy documents and interviews stressed the importance of developing improvement capability but faced definitional difficulties when articulating precisely, and consistently, what was meant by improvement capability. Furthermore, it was evident that the term was used inconsistently, boundaries between dimensions were blurred, and that it is a nebulous, ambiguous and subjective concept. For example, whilst interview participants were keen to stress the importance of organisational culture, it was acknowledged that is was a difficult concept to grasp, assess and for organisations to influence (see Table 4).

### Assessment data

The second theme highlights the challenges resulting from regulatory access to data, and identifies that existing and available data are used as proxy data sources in the absence of more appropriate data sources. For example, in Table 4 three data sources from Wales are highlighted within the dimension of employee commitment. These demonstrate the differences between assessment intentions and the data used during assessments. These examples, together with the other quotations in this dimension, also highlight how annually collected and available data from NHS staff surveys or locally produced staff turnover and vacancy data are used as indicators, despite policy intentions stressing employee contribution, ownership and engagement, rather than staffing numbers and proxy measures for employee commitment, such as vacancy rates and resilience.

### Assessment practices

Assessment practices for improvement capability are limited as there is variable understanding of improvement capability by regulatory agency staff. The variable understanding of improvement capability during assessments risks causing variation, bias and inconsistency through a self-confessed lack of knowledge. Interview data indicated that assessors were still learning how to assess for improvement capability and were unclear what evidence to seek. Most regulatory agencies were only beginning to focus on assessing and developing improvement capability in organisations. Thus, whilst policy documents and interview participants stressed this intent, further development of assessment practices, assessment processes and assessor understanding of improvement capability was still needed.

‘It is such a new area for our inspectors to, sort of, look at and look at it in this way.’ (Interview participant B, CQC)

‘We really need to understand the current state better and part of that is about understanding the capability of the workforce and the people that we’re actually going to be […] supporting […], but we’re not quite there yet systematically.’ (Interview participant C, HIS)

## Discussion

This research set out to explore how improvement capability is conceptualised and assessed by the UK healthcare regulatory agencies. The research study found that agencies aim to assess and develop improvement capability, but that two dimensions of improvement capability from a framework of eight dominate assessment: process improvement and learning, and strategy and governance. Other dimensions identified from the literature, such as employee commitment or organisational culture, are used less frequently during assessment, with some variation between agencies. Finally, in contrast to agency strategic messages to place the patient at the centre of their work, this research identifies that the area of lowest content frequency within policy documents and interviews was service-user focus, with only a small increase in the frequency of use in assessment documents.

Three themes emerge from this analysis of the assessment of improvement capability: conceptualisation, assessment data and assessment practices. A limited conceptualisation of improvement capability is operationalised by agencies when compared with the literature [12]. In line with other healthcare studies, for example, Brennan *et al*. [25], this research study finds that the assessment data used by agencies need further development to ensure that evidence collected does measure dimensions of improvement capability, which will strengthen the validity of the assessments. Furthermore, there are concerns that impact on measurement consistency, validity and reliability [9, 10], and the dependence on value-judgements of inspectors and surveyors [26], Finally, assessors need further skills, knowledge and guidance to assess across the broad range of improvement capability dimensions, in order to strengthen assessment in practice and reliability. These findings suggest that current assessments focus on dimensions which are easier to measure with more tangible evidence, such as the existence of a strategic plan, in contrast to the assessment of dimensions that are more ambiguous and difficult to assess. There are a number of existing validated models that could be used to strengthen assessments in these dimensions, for example, for organisational culture there are a number of existing models [27, 28], which could strengthen assessment effectiveness and resultant regulatory judgements. These findings suggest a regulatory intent that is still emerging, has been more difficult to implement in practice than anticipated, or that agency policies are not being implemented.

The themes provide suggestions for the development of agencies’ assessments of improvement capability within organisations. Regulatory agencies may use their assessments to determine and design their enforcement approach with organisations. Without a broader conceptualisation of improvement capability, enforcement approaches may not be designed to adequately meet organisational needs and be less effective. For example, agencies may inadequately or inaccurately assess an organisation’s capability to improve, instead indicating that external support is required, leading to the poor use of resources by both agencies and organisations, and negatively impacting morale. Finally, inaccurate assessments may undermine confidence in an organisation by local populations and stakeholders.

Building on these suggestions will assist agencies in meeting their aims of developing improvement capability through more effective assessment. This would enable agencies to ensure that organisations focus across all dimensions holistically. Furthermore, a broader conceptualisation would support increased attention on patient care across care pathways and between organisations, supporting the development of service integration through the use of the stakeholder and supplier, and service-user focus dimensions; it would also strengthen the reliability and validity of regulatory assessments.

Further research is required to support assessment and the subsequent tailoring of improvement support to organisations. This needs to be based on an understanding of their existing improvement capability, and to strengthen understanding about how improvement capability emerges or dissipates within organisations. Nevertheless, it is important to acknowledge the limitations of this research study, which largely relies on cross-sectional data from a regulatory agency perspective. Perspectives from assessed organisations would provide richer data and help to continue to build our understanding of improvement capability.

## Conclusions

This research study set out to consider how regulatory and improvement agencies assess improvement capability. Its analysis of policy documents, interviews and assessment reports shows that whilst all these agencies aim to assess and develop improvement capability within healthcare organisations, two out of eight dimensions of improvement capability dominate assessment. This may be due to the difficulty in operationalising the dimensions that comprise improvement capability due to measurement, knowledge and practice gaps.

Empirically, this research study has addressed a gap in the knowledge regarding the assessment of improvement capability, and the results provide a starting point for the development of which factors could be considered in the assessment of improvement capability. Better understanding and assessment of improvement capability will allow more tailored development approaches by regulatory and improvement agencies. This research study has highlighted the need for regulatory agencies to further conceptualise improvement capability in order to inform their assessment and subsequent development. This will strengthen agencies’ assessment, diagnosis and prediction of organisational performance trajectories, and support the development of more appropriate and effective regulatory interventions.
